# Multimodal Surgical Strategy with Preoperative Transcatheter Arterial Embolization Facilitates Safe and Controlled Resection of a Giant Intrathoracic Chronic Expanding Hematoma: A Case Report

**DOI:** 10.70352/scrj.cr.26-0369

**Published:** 2026-09-03

**Authors:** Taichiro Kawano, Hiroki Ebana, Daisuke Shimizu, Ryuhei Sakata, Aki Kobayashi

**Affiliations:** Department of Thoracic Surgery, Tokyo Metropolitan Bokutoh Hospital, Tokyo, Japan

**Keywords:** chronic expanding hematoma, intrathoracic hematoma, preoperative transcatheter arterial embolization, tuberculous pleuritis, chest wall plasty

## Abstract

**INTRODUCTION:**

Chronic expanding hematoma (CEH) is a slowly enlarging hematoma that persists for more than 1 month after the initial hemorrhagic event. Intrathoracic CEH is rare and often requires technically demanding surgery because of dense adhesions and the risk of massive intraoperative bleeding. We report a case of giant intrathoracic CEH managed using a surgical strategy that included preoperative selective transcatheter arterial embolization (TAE).

**CASE PRESENTATION:**

A 49-year-old man with a history of tuberculous pleuritis approximately 10 years earlier was referred to our hospital because of a gradually enlarging asymptomatic right intrathoracic mass. Contrast-enhanced CT revealed a 95-mm heterogeneous mass with peripheral enhancement, consistent with CEH. Selective embolization of the right 8th to 11th intercostal arteries was performed on the day before surgery. Through a posterolateral thoracotomy with resection of the 7th and 8th ribs, the hematoma was completely resected with partial resection of the right lower lobe. Additional procedures, including decortication, adhesiolysis, and chest wall plasty, were performed to facilitate lung re-expansion and reduce the residual thoracic cavity. Although prolonged postoperative air leakage required pleurodesis on POD 10, the patient was discharged on POD 12 without major complications. No recurrence has been observed during follow-up.

**CONCLUSIONS:**

Preoperative selective TAE, combined with an appropriate surgical strategy, may reduce intraoperative bleeding and enable safe and controlled radical resection of intrathoracic CEH. Careful preoperative planning, including bleeding control and optimization of the surgical approach, is essential for successful treatment.

## Abbreviations

CEA
: carcinoembryonic antigen
CEH
: chronic expanding hematoma
CYFRA
: cytokeratin 19 fragment
NSE
: neuron specific enolase
ProGRP
: pro-gastrin releasing peptide
SCC
: squamous cell carcinoma antigen
TAE
: transcatheter arterial embolization

## INTRODUCTION

CEH, first described by Reid et al.,^1)^ is defined as a hematoma that persists and gradually enlarges for more than 1 month after the initial hemorrhagic event. Its pathogenesis is thought to involve chronic inflammation induced by blood breakdown products and repeated bleeding from fragile capillaries within the fibrous capsule.^1,2)^

Intrathoracic CEH is a relatively rare condition that has been reported after tuberculous pleuritis, thoracic surgery, trauma, and chronic hemothorax.^3–5)^ Progressive enlargement of the lesion may result in compression of adjacent organs, respiratory symptoms, and occasionally life-threatening complications.^6,7)^ Complete surgical resection is generally considered the treatment of choice; however, surgery is often technically demanding because of dense adhesions, marked neovascularization, and the risk of massive intraoperative bleeding.^8–11)^

Although complete surgical resection is the treatment of choice, the optimal management strategy for giant intrathoracic CEH remains unclear because of its rarity.^8,9,12)^

Here, we report a case of giant intrathoracic CEH that developed after tuberculous pleuritis and was successfully treated using patient-specific surgical planning, including preoperative selective TAE, extended thoracotomy, and postoperative thoracic cavity management.

## CASE PRESENTATION

A 49-year-old man was referred to our hospital for evaluation of an enlarging right intrathoracic cystic lesion. He had undergone medical treatment for tuberculous pleuritis approximately 10 years earlier. He was asymptomatic.

On admission, his height was 183 cm and body weight was 57.3 kg. His blood pressure was 124/85 mmHg, pulse rate was 69 beats/min, respiratory rate was 16 breaths/min, and oxygen saturation was 98% on room air.

Laboratory tests showed a hemoglobin level of 14.8 g/dL, white blood cell count of 6200/μL, and C-reactive protein level of 0.12 mg/dL. Tumor marker evaluation revealed a mildly elevated CEA level of 8.1 ng/mL. The levels of SCC, CYFRA 21-1, NSE, and ProGRP were 1.1, 1.8, 13.0 ng/mL, and 71.3 pg/mL, respectively, with no other remarkable abnormalities. The mild elevation in CEA was considered nonspecific and unrelated to the lesion. Preoperative pleural fluid analysis showed bloody effusion with a cell count of 2280/μL (83% polymorphonuclear cells and 17% mononuclear cells), total protein 7.2 g/dL, albumin 4.5 g/dL, lactate dehydrogenase 269 IU/L, pH 7.54, glucose 87 mg/dL, adenosine deaminase 19.9 U/L, and hemoglobin 14.9 g/dL. Cytological examination showed no malignant cells.

Electrocardiography showed sinus rhythm with a heart rate of 60 beats/min. Pulmonary function testing showed a vital capacity of 4.20 L (%VC 86.2%), forced expiratory volume in 1 second of 3.10 L, and FEV1.0% of 75.9%.

**Fig. 1:**
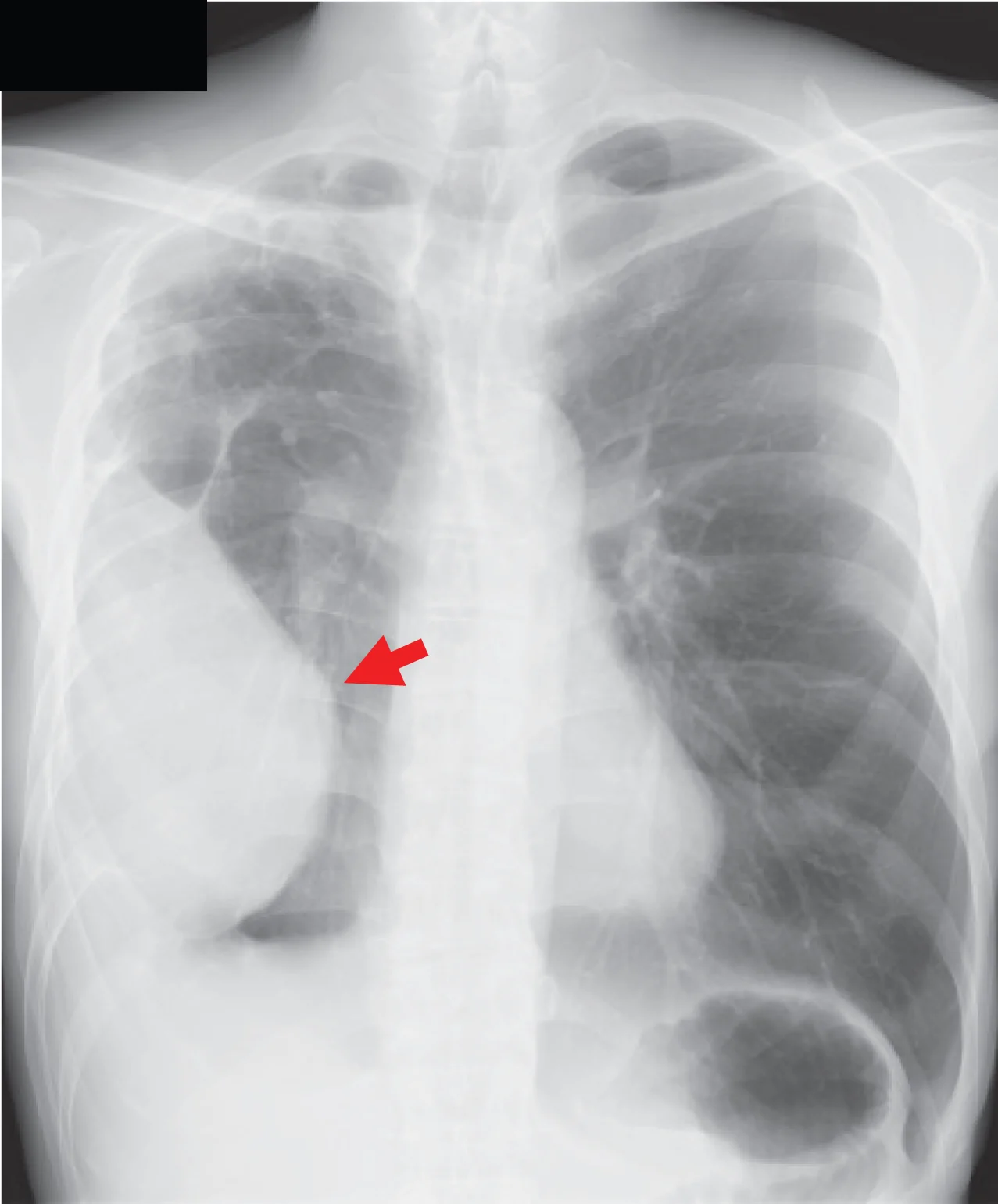
Chest radiograph findings. A chest radiograph showed a mass shadow in the right middle and lower lung fields (arrow).

**Fig. 2:**
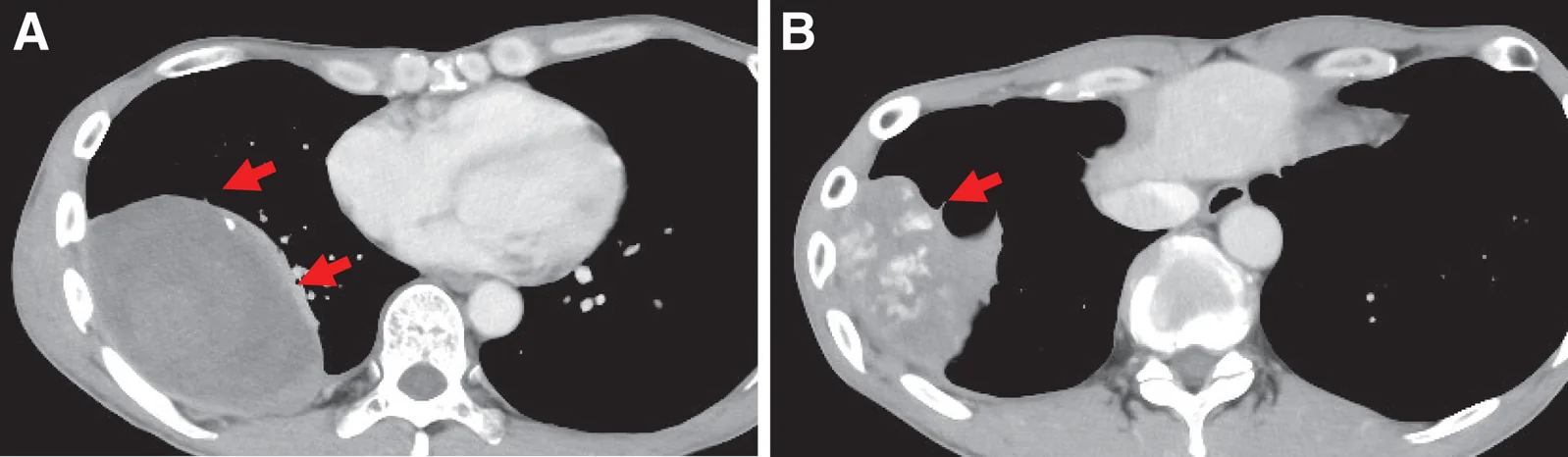
Contrast-enhanced CT findings. (**A**) The early-phase image showed predominant peripheral enhancement with heterogeneous internal attenuation (arrows). (**B**) The delayed-phase image showed progressive enhancement of the lesion (arrow).

Chest radiography demonstrated a mass shadow in the right middle to lower lung field (**Fig. 1**). Contrast-enhanced CT revealed a 95-mm right intrathoracic mass with heterogeneous internal density and peripheral enhancement in the early phase, findings consistent with CEH (**Fig. 2A**). Progressive internal enhancement was also observed (**Fig. 2B**).

**Fig. 3:**
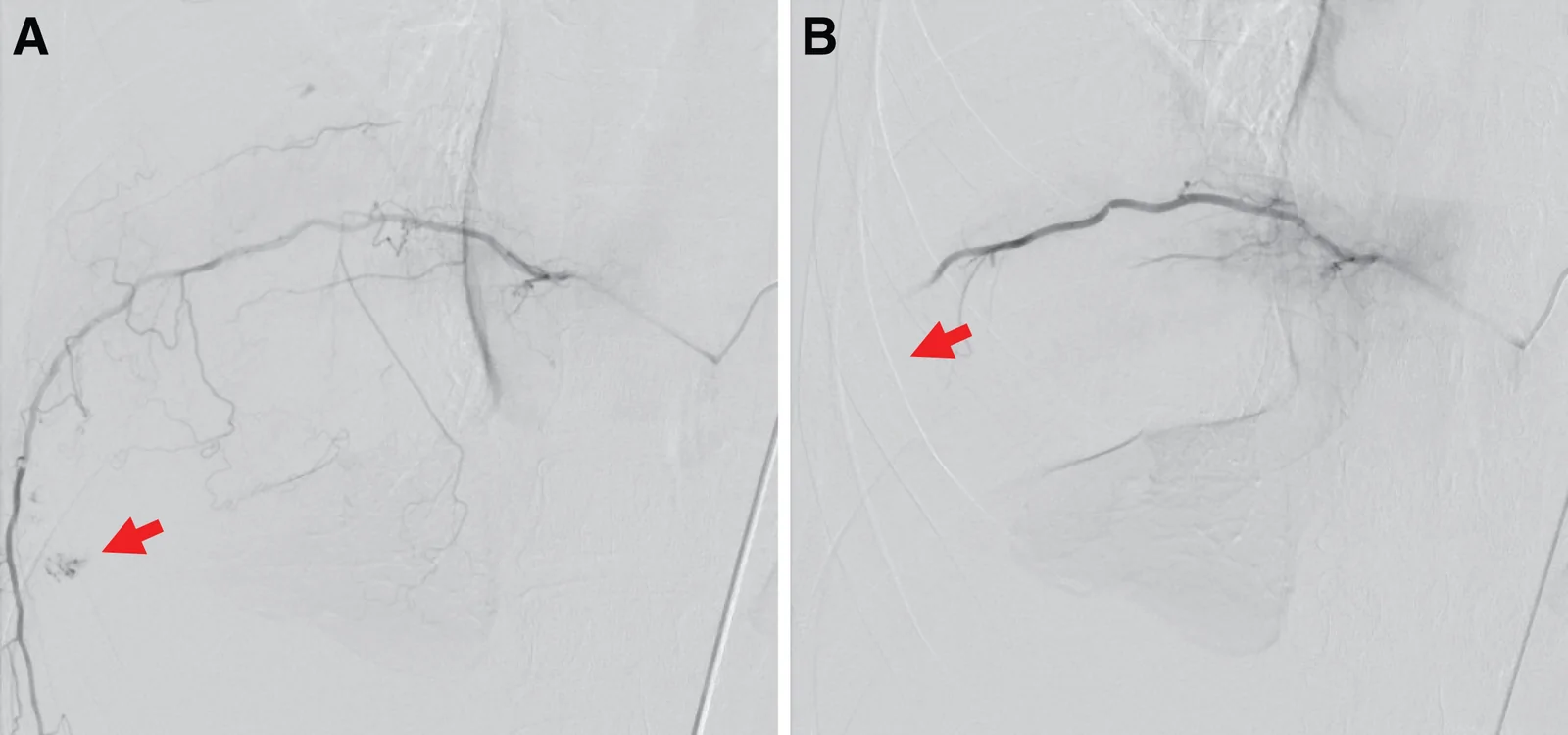
Selective angiography and embolization of the right 9th intercostal artery. (**A**) Selective angiography of the right 9th intercostal artery before embolization demonstrated focal extravasation around the mass (arrow). (**B**) Angiography after embolization showed disappearance of the extravasation (arrow).

Selective angiography of the right 8th and 9th intercostal arteries demonstrated peripheral extravasation, and embolization with gelatin sponge was subsequently performed (**Fig. 3**). Although obvious extravasation was not identified in the right 10th and 11th intercostal arteries, embolization was also performed based on a combination of angiographic findings and preoperative CT assessment of potential feeding vessels.

**Fig. 4:**
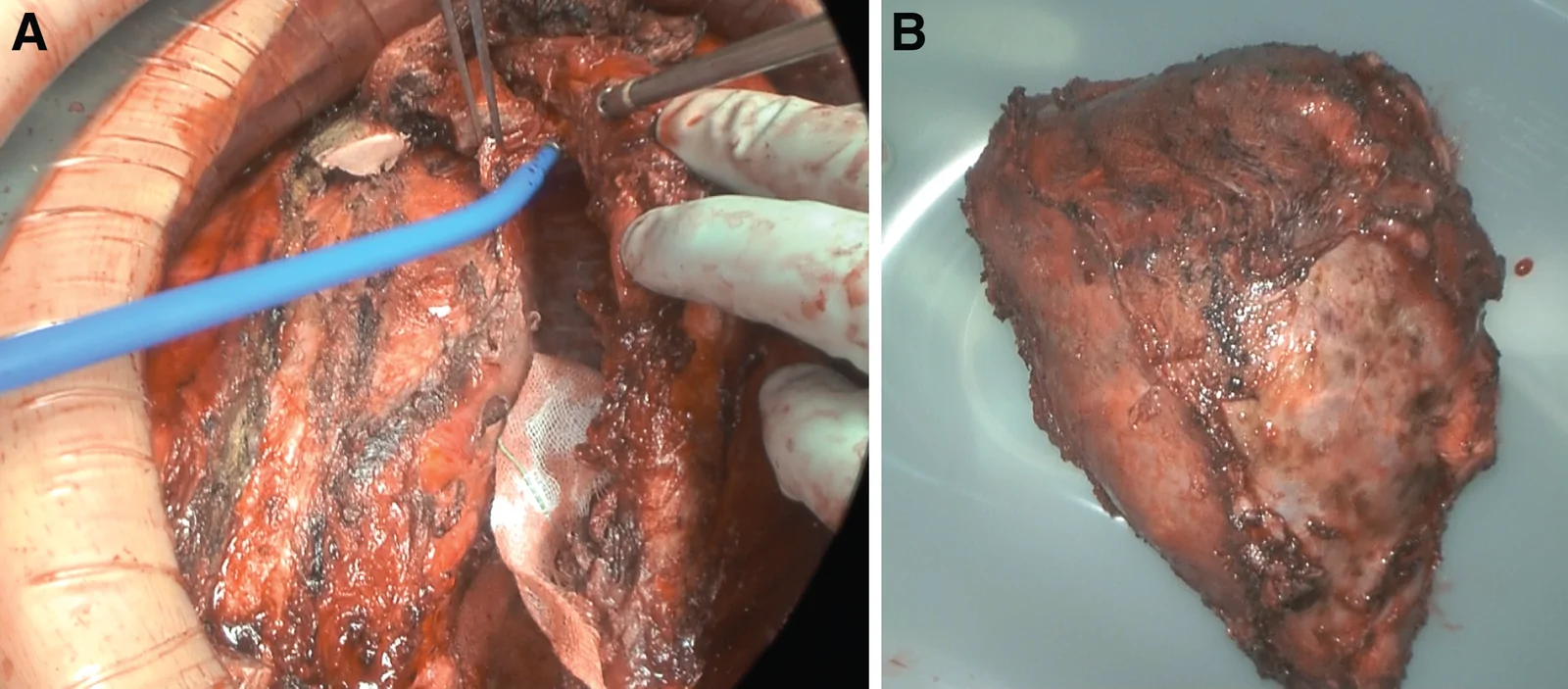
Intraoperative findings and resected specimen. (**A**) Intraoperative view after resection of the 7th and 8th ribs, showing the exposed hematoma and adequate operative field. (**B**) The resected specimen showed a thick fibrous capsule surrounding the hematoma.

Surgery was performed under general anesthesia with 1-lung ventilation in the left lateral decubitus position. A posterolateral thoracotomy approximately 40 cm in length was made from the subscapular region to the anterior chest to secure adequate exposure. The latissimus dorsi muscle was preserved, and the serratus anterior muscle was retracted. Thoracotomy was performed through the 6th intercostal space to enter the thoracic cavity. Extensive intrathoracic adhesions were present, and the lung was fragile. The surface of the hematoma was white and firm, and the border between the hematoma and the lung was unclear. The 7th and 8th ribs were resected to secure the operative field (**Fig. 4A**). The hematoma was dissected mainly on the hematoma side using energy devices, a ball electrode, and electrocautery. A diaphragmatic injury occurred during dissection and was repaired using 3-0 PROLENE polypropylene suture (SH-1; Ethicon, Somerville, NJ, USA). The hematoma was ultimately removed with wedge resection of the right lower lobe (**Fig. 4B** and **Video 1**), demonstrating the en bloc resection. Because re-expansion of the remaining lower lobe was poor due to long-term compression, decortication of the thickened visceral pleura of the lower lobe was performed to facilitate lung re-expansion and reduce the residual pleural space. In addition, adhesiolysis of the upper and middle lobes and fissure formation between the middle and lower lobes were carried out. Air leakage was treated with soft coagulation and 10 mL of fibrin glue (5 mL of BOLHEAL [KM Biologics, Kumamoto, Japan] and 5 mL of Beriplast P [CSL Behring, Tokyo, Japan]). To reduce the residual thoracic cavity, the 9th rib was transected for chest wall plasty. Two 24-Fr chest drains were placed, and the chest wall defect was reinforced with Bard Soft Mesh (Davol, Warwick, RI, USA) (**Video 2**), demonstrating decortication and chest wall plasty. The operative time was 5 h 24 min, and the intraoperative blood loss was 514 mL. No blood transfusion was required.

**Fig. 5:**
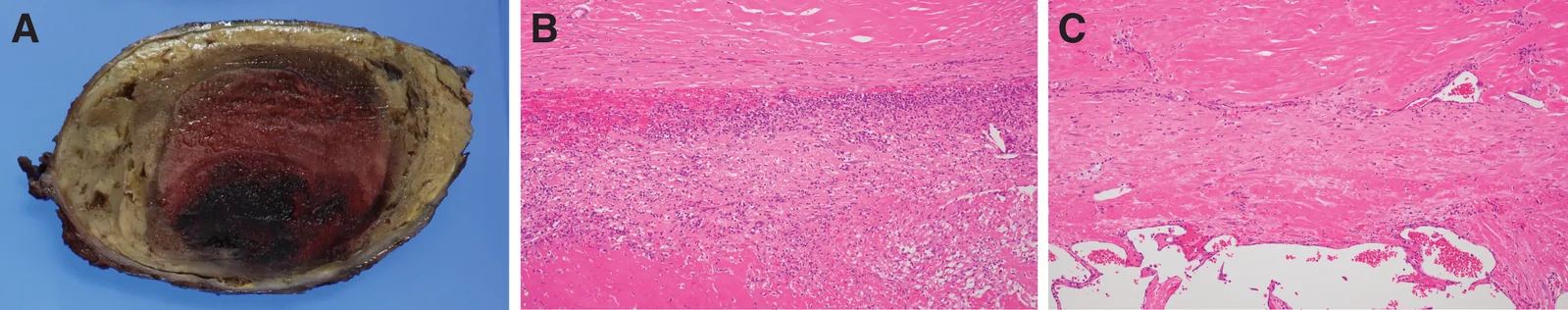
Pathological findings. (**A**) Gross appearance of the resected specimen showing a thick fibrous capsule and mixed old and new hematoma. (**B**) Histological findings showing relatively fresh hemorrhage and inflammatory cell infiltration beneath the hyalinized fibrous capsule (hematoxylin and eosin staining, ×100). (**C**) Neovascularization within the fibrous capsule (hematoxylin and eosin staining, ×200). These findings were consistent with CEH. CEH, chronic expanding hematoma

Pathological examination showed that the resected mass had a thick fibrous capsule and contained mixed old and new hematoma. Beneath the hyalinized fibrous capsule, relatively fresh hemorrhage and inflammatory cell infiltration were observed. Neovascularization was also identified, and these findings were consistent with CEH (**Fig. 5**).

**Fig. 6:**
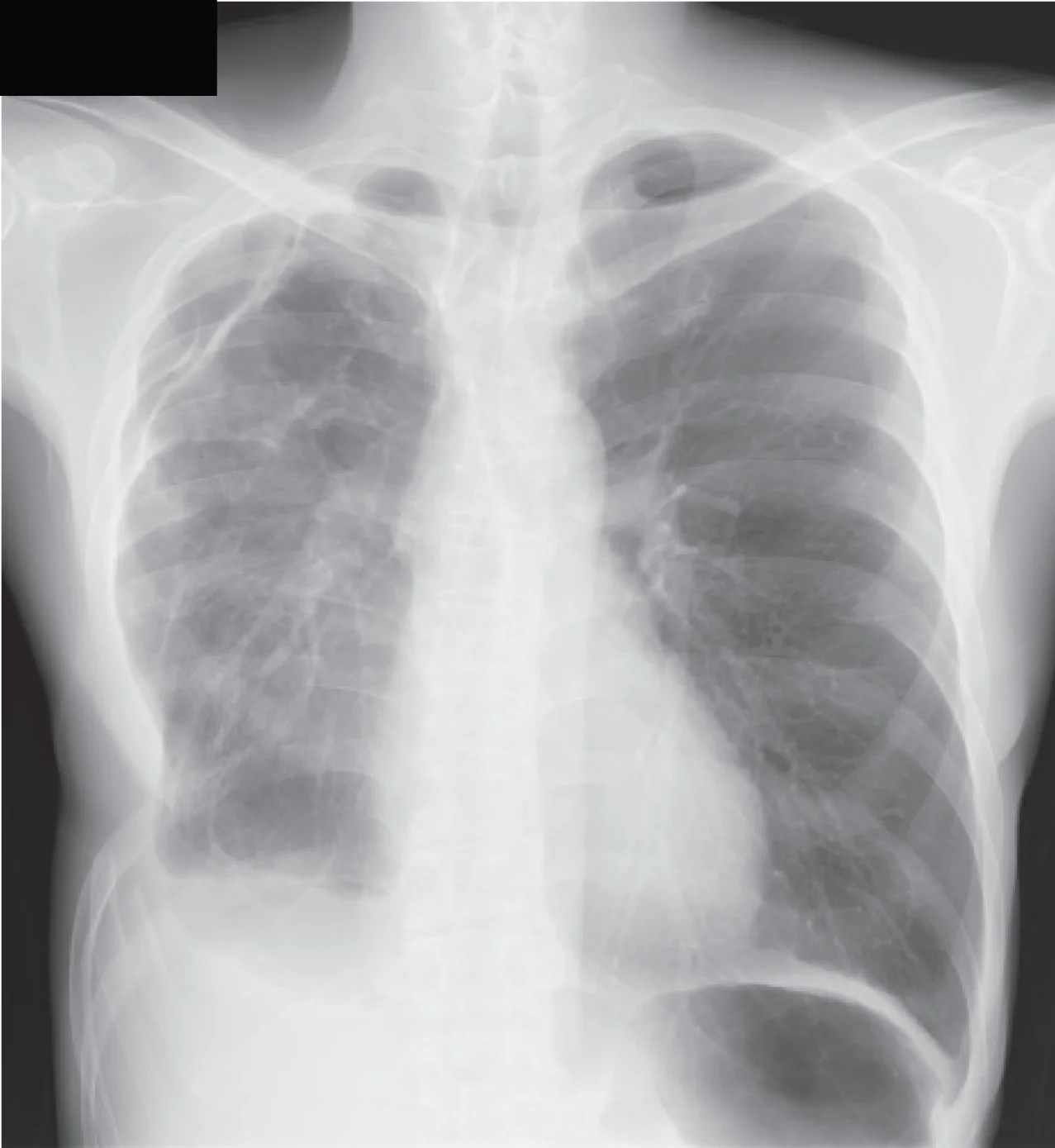
Chest radiograph at 6 months after surgery. Chest radiograph obtained 6 months after surgery showed good re-expansion of the remaining right lung, with no evidence of recurrence.

Postoperatively, prolonged air leakage occurred, and pleurodesis using OK-432 (PICIBANIL; Chugai Pharmaceutical, Tokyo, Japan) was performed on POD 10. The chest drains were removed on POD 11, and the patient was discharged on POD 12. At 6 months after surgery, imaging showed good re-expansion of the remaining lung with no abnormalities of the repaired diaphragm (**Fig. 6**).

## DISCUSSION

Intrathoracic CEH is a rare condition in which complete surgical resection is often technically demanding because of dense adhesions, abundant neovascularization, and the risk of massive intraoperative bleeding.^8–11,13)^ The present case demonstrates that management of giant intrathoracic CEH requires not only complete resection but also careful patient-specific surgical planning, including preoperative bleeding control, adequate operative exposure, and postoperative thoracic cavity management.

Based on this patient-specific surgical plan, preoperative selective angiography demonstrated extravasation from the right 8th and 9th intercostal arteries. In addition, the right 10th and 11th intercostal arteries were considered potential feeding vessels based on contrast-enhanced CT findings, and embolization was performed accordingly. The preoperative diagnosis was based on the patient's history of tuberculous pleuritis, gradual enlargement of the lesion over approximately 5 years, and CT findings demonstrating a heterogeneous mass with suspected feeding vessels arising from the intercostal arteries. Although malignant tumors remained in the differential diagnosis, intrathoracic CEH was considered the most likely diagnosis. Because progressive enlargement and substantial intraoperative bleeding were anticipated regardless of the final pathological diagnosis, selective TAE was performed before surgery. The diagnosis was ultimately confirmed by pathological examination of the resected specimen.

The angiographic findings observed in the present case suggest that neovascularization from the intercostal arteries may have contributed to persistent bleeding and gradual enlargement of the hematoma. Although this mechanism cannot be confirmed from a single case, the angiographic findings support the hypothesis that collateral vessels arising from the intercostal arteries may play an important role in maintaining the vascularity of intrathoracic CEH. Although TAE is not curative as a standalone treatment, it may serve as a useful adjunct for reducing intraoperative bleeding and facilitating dissection.^12)^ In the present case, intraoperative blood loss was 514 mL, and no blood transfusion was required. While the relatively low blood loss observed in this case may suggest a potential benefit of preoperative TAE in combination with meticulous surgical planning, the contribution of TAE cannot be isolated from other surgical factors, including adequate exposure and meticulous dissection. Moreover, TAE should be regarded as an adjunctive procedure rather than definitive treatment, as incomplete embolization or collateral circulation may limit its effectiveness. Therefore, careful preoperative vascular evaluation and appropriate embolization planning remain essential.

From a surgical perspective, adequate operative exposure was considered essential to achieve controlled dissection without capsular rupture or uncontrolled bleeding.^4,8)^ Because extensive pleural adhesions related to previous tuberculous pleuritis and the large size of the hematoma prevented adequate visualization through a standard thoracotomy, the surgical approach was extended to obtain sufficient working space. This strategy facilitated en bloc resection while minimizing the risk of uncontrolled bleeding and capsular rupture. Intraoperative bleeding control was further supported by the use of an energy device, which helped maintain a stable operative field during dissection.

Another critical issue in this case was poor re-expansion of the residual lung due to long-term compression. Decortication and additional procedures to facilitate lung re-expansion were performed to optimize postoperative lung expansion and reduce the residual pleural space. Management of the residual thoracic cavity was also an important component of the surgical strategy. In intrathoracic CEH, successful treatment requires not only removal of the lesion but also optimization of postoperative thoracic configuration and lung re-expansion.^9,13)^ Thoracic cavity reduction was performed to minimize the residual dead space. Because satisfactory lung re-expansion was anticipated after decortication and reduction of the thoracic cavity, rigid chest wall reconstruction was not considered necessary. Instead, mesh reinforcement was selected primarily to prevent postoperative lung herniation. The mesh has been routinely used at our institution for chest wall reconstruction with satisfactory clinical outcomes and was therefore considered an appropriate reconstructive material in the present case. Furthermore, the latissimus dorsi muscle was intentionally preserved to maintain future reconstructive options should infection or other reconstruction-related complications occur. Omental flap transposition was not performed because the residual dead space was considered acceptable after thoracic cavity reduction and satisfactory lung re-expansion was expected.

Failure to address the residual thoracic cavity may lead to persistent dead space, impaired lung re-expansion, and postoperative complications. Although prolonged air leakage occurred postoperatively and required pleurodesis using OK-432 (picibanil), satisfactory lung re-expansion was ultimately achieved, and no recurrence has been observed.

The educational value of the present case does not lie in any single operative technique, as all procedures performed, including TAE, extended thoracotomy, rib resection, decortication, thoracic cavity reduction, and mesh reinforcement, are established surgical techniques. Rather, this case highlights the importance of patient-specific surgical planning that integrated these procedures according to the anticipated intraoperative findings and postoperative thoracic configuration. This individualized approach enabled complete resection with limited blood loss, preservation of the remaining lung, and satisfactory postoperative lung re-expansion.

This report has several limitations. First, it describes a single case, and the precise contribution of preoperative TAE to reducing blood loss cannot be quantified. Second, the optimal target vessels and timing of embolization remain to be established. Nevertheless, this case highlights the importance of patient-specific surgical planning in the management of giant intrathoracic CEH.

## CONCLUSIONS

This case demonstrates that careful patient-specific surgical planning, incorporating established surgical techniques tailored to the individual pathological and anatomical characteristics, can facilitate effective surgical management of giant intrathoracic CEH. Careful preoperative planning, including bleeding control and optimization of the surgical approach, is essential for successful treatment.

## SUPPLEMENTARY MATERIALS

Video 1 Surgical procedure from skin incision to complete resection of a giant intrathoracic CEH (2× speed). A posterolateral thoracotomy was performed, and dense intrathoracic adhesions were carefully dissected using electrocautery and energy devices. The 7th and 8th ribs were transected to secure adequate operative exposure. Despite firm adhesions between the hematoma and surrounding structures, including the lung, chest wall, and diaphragm, the lesion was dissected mainly along the hematoma side. Intraoperative bleeding was effectively controlled using soft coagulation, enabling en bloc resection without rupture of the hematoma, demonstrating controlled resection under effective bleeding management.

Video 2 Post-resection management focusing on lung re-expansion and optimization of the residual thoracic cavity (2× speed). After repair of the diaphragmatic injury, adhesiolysis of the middle and lower lobes and decortication of the lower lobe were performed to facilitate lung re-expansion. Air leaks were controlled using soft coagulation and fibrin glue. To reduce residual thoracic dead space, the 9th rib was transected, followed by chest wall reconstruction using mesh.

## DECLARATIONS

### Availability of data and materials

All data generated or analyzed during this study are included in this published article.
